# Supplementary material for: MetaQTL: a package of new computational methods for the meta-analysis of QTL mapping experiments
Source: BMC Bioinformatics. 2007 Feb 8;8:49. doi: 10.1186/1471-2105-8-49 (PMC1808479; doi:10.1186/1471-2105-8-49)
Supplement: Additional File 7 — MetaQTL Package : jar file and tutorial. This Zip archive contains both the MetaQTL JAR file and the files of the tutorial. [file 1471-2105-8-49-S7.zip › org.inra.metaqtl/doc/org/metaqtl/bio/entity/Population.html]

Population


|  |  |  |
| --- | --- | --- |
| **Overview** \| **Package** \| **Class** \| **Use** \| **Tree** \| **Deprecated** \| **Index** \| **Help** | |  |
| **PREV CLASS**   **NEXT CLASS** | **FRAMES**    **NO FRAMES**     **All Classes** |  |
| SUMMARY: NESTED \| FIELD \| CONSTR \| METHOD | DETAIL: FIELD \| CONSTR \| METHOD |  |


---


## org.metaqtl.bio.entity Class Population

```
java.lang.Object
  org.metaqtl.bio.entity.BioEntity
      org.metaqtl.bio.entity.BioEntityContainer
          org.metaqtl.bio.entity.IndividualContainer
              org.metaqtl.bio.entity.Population
```

**All Implemented Interfaces:**: IBioAdaptable, IBioEntity, IBioPopulation

**Direct Known Subclasses:**: CrossPopulation

---

``` public class Population extends IndividualContainer implements IBioPopulation ```

Class Description Here

**Author:**
:   Jean-Baptiste Veyrieras

---

| **Field Summary** | |
| --- | --- |
| `protected  int` | `generation` |
| `IBioIndividual[]` | `parents` |
| `protected  int` | `size` |

**Fields inherited from class org.metaqtl.bio.entity.BioEntityContainer**

`entities`

**Fields inherited from class org.metaqtl.bio.entity.BioEntity**

`name, parent, properties`


**Constructor Summary**

`Population()`

`Population(java.lang.String name,
IBioEntity parent)`


| **Method Summary** | |
| --- | --- |
| `IBioAdapter` | `getBioAdapter()` |
| `int` | `getGeneration()` |
| `int` | `getIndividualNumber()` |
| `IBioIndividual[]` | `getParents()` |
| `int` | `getSize()`             Returns the size of the population. |
| `int` | `getType()`             There are 2 main class of entities. |
| `static Population` | `newCrossPopulation(int type, java.lang.String name, IBioEntity parent)` |
| `void` | `setGeneration(int generation)` |
| `void` | `setSize(int size)`             Set the population size. |

**Methods inherited from class org.metaqtl.bio.entity.IndividualContainer**

`addIndividual, getIndividual, individuals, removeIndividual`

**Methods inherited from class org.metaqtl.bio.entity.BioEntityContainer**

`addEntity, entities, entityNumber, getEntity, removeEntity`

**Methods inherited from class org.metaqtl.bio.entity.BioEntity**

`getName, getParent, getProperties, newBioEntity, setName, setProperties`

**Methods inherited from class java.lang.Object**

`clone, equals, finalize, getClass, hashCode, notify, notifyAll, toString, wait, wait, wait`

**Methods inherited from interface org.metaqtl.bio.IBioPopulation**

`addIndividual, getIndividual, individuals, removeIndividual`

**Methods inherited from interface org.metaqtl.bio.IBioEntity**

`getName, getParent, getProperties, setName, setProperties`

**Field Detail**

### size

```
protected int size
```

---


### generation

```
protected int generation
```

---


### parents

```
public IBioIndividual[] parents
```


**Constructor Detail**

### Population

```
public Population()
```

---


### Population

```
public Population(java.lang.String name,
                  IBioEntity parent)
```

**Parameters:**: `name` -: `parent` -


**Method Detail**

### newCrossPopulation

```
public static Population newCrossPopulation(int type,
                                            java.lang.String name,
                                            IBioEntity parent)
```

---


### getType

```
public int getType()
```

:   **Description copied from interface: `IBioEntity`**
:   There are 2 main class of entities. The first one deals with population
    biological entity, i.e population itself and individuals. The second class
    is a representation of microscopic biological entity from genome container
    to alleles.

    :   **Specified by:**: `getType` in interface `IBioEntity` **Specified by:**: `getType` in class `IndividualContainer`

---


### getIndividualNumber

```
public int getIndividualNumber()
```

:   **Specified by:**: `getIndividualNumber` in interface `IBioPopulation` **Specified by:**: `getIndividualNumber` in class `IndividualContainer`

---


### setSize

```
public void setSize(int size)
```

:   Set the population size.

    :   **Parameters:**: `size` - The size to set.

---


### getSize

```
public int getSize()
```

:   Returns the size of the population.

    :   **Returns:**

---


### getBioAdapter

```
public IBioAdapter getBioAdapter()
```

:   **Specified by:**: `getBioAdapter` in interface `IBioAdaptable`

---


### getGeneration

```
public int getGeneration()
```

:   **Returns:**: Returns the generation.

---


### setGeneration

```
public void setGeneration(int generation)
```

:   **Parameters:**: `generation` - The generation to set.

---


### getParents

```
public IBioIndividual[] getParents()
```


---


|  |  |  |
| --- | --- | --- |
| **Overview** \| **Package** \| **Class** \| **Use** \| **Tree** \| **Deprecated** \| **Index** \| **Help** | |  |
| **PREV CLASS**   **NEXT CLASS** | **FRAMES**    **NO FRAMES**     **All Classes** |  |
| SUMMARY: NESTED \| FIELD \| CONSTR \| METHOD | DETAIL: FIELD \| CONSTR \| METHOD |  |


---
